# The relationship between serum manganese concentration with all-cause and cause-specific mortality: a retrospective and population-based cross-sectional study

**DOI:** 10.1186/s12872-024-03872-5

**Published:** 2024-04-27

**Authors:** Jianyun Ou, Yunfei Sun, Jie Tong, Weihong Tang, Genshan Ma

**Affiliations:** Department of Cardiology, Zhongda Hospital, Southeast University, 87 Dingjiaqiao, Nanjing, 210009 P.R. China

**Keywords:** All-cause mortality, Cardiovascular disease mortality, Cancer-related mortality, Exposure, Manganese, Risk assessment

## Abstract

**Background:**

The study aimed to explore the association between manganese concentration and all-cause, cardiovascular disease (CVD)-related, and cancer-related mortality in the general population of the United States.

**Methods:**

We integrated the data from the National Health and Nutrition Examination Survey from 2011 to 2018. A total of 9,207 subjects were selected based on the inclusion and exclusion criteria. The relationship between manganese concentration and all-cause, CVD-related, and cancer-related mortality was analyzed by constructing a Cox proportional hazard regression model and a restricted cubic spline (RCS) plot. Additionally, subgroup analyses stratified by age, sex, race/ethnicity, hypertension, diabetes mellitus (DM), chronic heart disease, chronic heart failure, angina pectoris, heart attack, stroke, and BMI were further performed.

**Results:**

In the full adjusted model, compared with the lowest quartile, the adjusted hazard ratios with 95% confidence intervals (CIs) for all-cause, CVD-related, and cancer-related mortality across manganese quartiles were (1.11 (0.87,1.41), 0.96 (0.74, 1.23), and 1.23 (0.96, 1.59); *P*-value for trend =0.041), (0.86 (0.54, 1.37), 0.87 (0.55, 1.40), and 1.07 (0.67, 1.72); *P*-value for trend =0.906), and (1.45 (0.92, 2.29), 1.14 (0.70, 1.88), and 1.26 (0.75, 2.11); *P*-value for trend =0.526), respectively. The RCS curve shown a U-shaped association between manganese concentration and all-cause mortality and CVD-related mortality (*P*-value for nonlinear <0.05). However, there was an increase and then a decrease in the link between manganese concentration and cancer-related mortality (*P*-value for nonlinear <0.05). Manganese exposure was positively correlated with sex (correlation coefficient, r =0.19, *P*-value <0.001) and negatively correlated with age (correlation coefficient, *r* =-0.11, *P*-value <0.001) and serum creatinine (correlation coefficient, *r* =-0.12, *P*-value <0.001), respectively.

**Conclusions:**

Our findings suggest that elevated serum manganese concentrations are associated with all-cause and CVD-related mortality in the U.S. population and that maintenance of serum manganese between 8.67-9.23 µg/L may promote public health.

## Introduction

According to the global death statistics from the World Health Organization (WHO), cardiovascular diseases (CVD) account for more than 30% of all deaths worldwide [1]. Meanwhile, cancer also has the highest mortality rate in the world. In 2012, a total of 8.2 million cancer-related deaths were reported worldwide [2]. In addition to the use of tobacco, the consumption of alcohol, an unhealthy diet, and a lack of physical activity, environmental pollution is strongly associated with the prevalence of CVD and cancer [3].

As a result of their toxicity, long-term chronic exposures to heavy metals are a threat to human health [4]. The relationship between exposure to heavy metals and CVD-related and cancer-related diseases has been reported in some studies [5–7]. Manganese is an essential trace element that is involved in lipid metabolism and carbohydrate synthesis [8]. It serves as a regulator for the growth and reproduction of the body and the formation of tissues [9]. Manganese exists in a variety of oxidation states in the environment and is a cofactor for a variety of enzymes, and divalent manganese easily replaces Zn (II), Fe (II), and Co (II) in the metal active site of enzymes while often retaining its biological function [10]. The main sources of human ingestion of manganese are food and drinking water. Environmental exposure, including skin contact and air pollution, are the secondary routes. At the appropriate concentration, manganese acts as an antioxidant and is necessary for the synthesis of superoxide dismutase [11]. When the manganese concentration in the environment is more than 50 ng/m^3^, it is considered toxic exposure, which will endanger human health [12]. The 5th Chinese National Nutrition and Health Survey (2010–2012), exploring the correlation between manganese and metabolic syndrome (Mets), has found that higher manganese intake is associated with a reduced risk of Mets in men but an increased risk in women [13]. Shengjue Xiao et al. revealed that manganese levels are negatively correlated with the prevalence of CVD in a nation-wide study of older U.S. adults [8]. In addition, Maya Spaur and his team found that higher blood manganese was positively associated with liver steatosis [14]. Even though manganese is listed as a toxic heavy metal, its role as an antioxidant to benefit the heart cannot be overlooked [15]. Additionally, inadequate dietary manganese intake has been linked to adverse health effects such as diabetes, metabolic syndrome, and cancer [16]. It is therefore of great importance to find an appropriate manganese concentration in order to reduce all-cause, CVD, and cancer-related mortality. According to existing research, the association between manganese and all-cause, CVD-related, and cancer-related mortality in adults has not been fully examined. The National Health and Nutrition Examination Survey (NHANES) is a nationally representative study of the U.S. population. The NHANES uses a dynamic, multi-stage probability sampling design to obtain detailed information about the health and nutrition of the U.S. population. It has the advantages of a large sample size and good generalizability to U.S. populations. Thus, the purpose of this study was to investigate the relationship between serum manganese concentration and all-cause, CVD-related, and cancer-related mortality by integrating NHANES data from 2011 to 2018 and to provide guidance for clinical decision-making.

## Material and methods

### Study population

The cross-sectional NHANES database is a survey of the non-institutionalized general U.S. population that uses a dynamic, multistage probability sampling design to provide multitudinous information about the nutrition and health of the U.S. general population [17]. To investigate the correlation between manganese exposure and all-cause, CVD-related, and cancer-related mortality, we used data from the NHANES from 2011 to 2018. Among the 42,841 subjects in the total sample admitted, a total of 5,104 subjects with missing data on manganese were removed. Furthermore, we excluded subjects who missed data on all-cause, CVD-related, and cancer-related mortality (*n* =26749). To ensure the reliability of the results, missing covariates were also removed (*n* =6885). Ultimately, there were 9207 subjects that were included in our analysis. The detailed survey design, methodology, and data can be acquired on the NHANES website (https://www.cdc.gov/nchs/nhanes/).

### Manganese measurements

A blood sample was taken from subjects after they had been verified to be free of background contamination. Whole blood samples are processed, stored, and shipped to the National Center for Environmental Health, and Centers for Disease Control and Prevention, Atlanta, GA for analysis. Whole blood samples were analyzed for manganese content using mass spectrometry. The lower limit of detection and higher limit of detection for manganese in this study were 2.21 μg/L and 65.5 ug/L, respectively. A normal manganese level in human blood is between 4 and 15 μg/L [12]. Information regarding the experimental details can be found at https://wwwn.cdc.gov/nchs/data/nhanes/2015-2016/labmethods/PBCD_I_met.pdf.

### Covariates

The following covariates were taken into account in the analysis: age, sex (man/women), race/ethnicity (Mexican American, other Hispanic American, non-Hispanic black, non-Hispanic white, and other races), education level (less than high school/high school/more than high school), family poverty-income ratio (PIR), marital status (have a partner, no partner and unmarried), the complication of hypertension, diabetes mellitus (DM), chronic heart failure (CHF), chronic heart disease (CHD), angina pectoris, heart attack, and stroke, mean energy intake, body mass index (BMI), waist circumference, smoker (never, former, and now), alcohol user (never, former, mild, moderate, and heavy), physical activity (PA; never, moderate, both, and vigorous), systolic blood pressure (SBP), and diastolic blood pressure (DBP). In addition, laboratory tests provided information on hemoglobin (Hb), glucose, high-density lipoprotein cholesterol (HDL-C), total cholesterol (TC), triglyceride (TG), blood urea nitrogen (BUN), uric acid (UA), and serum creatinine (Scr). Details of the NHANES procedures can be found at https://wwwn.cdc.gov/nchs/nhanes/Default.aspx.

### All-cause, CVD-related, and cancer-related mortality

All-cause mortality was the primary outcome and was defined as mortality due to any cause during follow-up. Follow-up commenced on the baseline examination date. CVD and cancer-related mortality were considered the secondary and third outcomes, respectively. CVD mortality included death due to diseases of heart (I00-I09, I11, I13, I20-I51) and cerebrovascular diseases (I60-I69). Cancer-related mortality included death due to malignant neoplasms (C00-C97). The National Center for Health Statistics provides a detailed explanation of the linkage method and analytic guidelines on the data linkage webpage.

### Statistical analysis

R version 3.6.4 (R Foundation for Statistical Computing, Vienna, Austria) and SPSS version 22.0 (SPSS Inc., Chicago, IL, USA) were used to perform the statistical analyses. The *P*-value < 0.05 was considered statistically significant. Based on the NHANES sample weights, all estimates were calculated. The continuous variables were described by using the means ± standard deviation (SD), and the categorical variables were expressed as numbers (percentage, %). The differences between different groups were calculated using weighted Student’s t-test (continuous variables) or weighted chi-square test (categorical variables). Pearson’s correlation coefficients were calculated between manganese exposure and sociodemographic and cardiovascular outcomes. Due to its skewed distribution, manganese concentration was transformed using the log2 transformation. The association between manganese quartile and mortality (all-cause, CVD-related, and cancer-related) was studied using the Cox proportional hazard regression model. Firstly, model 1 was adjusted for age and sex. In model 2, we further adjusted for other potential confounders, including age, sex, education level, race/ethnicity, material status, family PIR, smoker, alcohol user, the complication of hypertension, and DM. Based on Model 2, the full adjustment was made for SBP, DBP, BMI, waist circumference, PA, mean energy intake, hemoglobin, fast glucose, BUN, UA, Scr, TC, TG, and HDL-C (Model 3). In the study, to detect multicollinearity, the variance inflation factor (VlF) analysis is applied to evaluate all the candidate variables [18]. VIF values are variance inflation factors that measure the severity of multicollinearity. It is generally believed that the VIF value is greater than 10, and there is a multicollinearity problem. Finally, subgroup analyses for the associations of manganese with all-cause, CVD-related, and cancer-related mortality were conducted based on age, sex, race/ethnicity, hypertension, DM, CHD, CHF, angina pectoris, heart attack, stroke, and BMI [19].

## Results

### Baseline characteristics

Table 1 displayed the weighted demographic and medical characteristics of participants by quartile of blood manganese concentration. Our study involved a total of 9,207 subjects. The subjects were categorized according to the manganese quartile (Q1: 1.570–7.415 μg/L; Q2: 7.416–9.230 μg/L; Q3: 9.231–11.555 μg/L; and Q4: 11.556–57.770 μg/L). All-cause, CVD-related, and cancer-related mortality were found to be present in 5.5%, 1.6%, and 1.4% of the study population, respectively. Age, race/ethnicity, sex, smoking status, family PIR, alcohol status, BMI, mean energy intake, waist circumference, SBP, Hb, BUN, UA and Scr differed significantly across quartiles of manganese (*P*-value <0.05).

**Table 1:** Weighted characteristics of the study population based on Manganese quartiles

Manganese (ug/L)	Total (*n*=9207)	Q1 (*n*=2302)	Q2 (*n*=2313)	Q3 (*n*=2290)	Q4 (*n*=2302)	*P*-value
Age, years	47.90 ± 0.39	49.74 ± 0.58	48.33 ± 0.55	47.18 ± 0.58	46.03 ± 0.46	< 0.001
Sex, %						< 0.001
Male	4563 (49.6%)	1419 (15.4%)	1275 (13.8%)	1106 (12.0%)	763 (8.3%)	
Female	4644 (50.4%)	913 (9.9%)	1116 (12.1%)	1229 (13.3%)	1386 (15.1%)	
Race, %						< 0.001
Mexican American	1098 (11.9%)	176 (1.9%)	260 (2.8%)	302 (3.3%)	360 (3.9%)	
Other Hispanic	910 (9.9%)	195 (2.1%)	250 (2.7%)	246 (2.7%)	219 (2.4%)	
Non-Hispanic Black	2063 (22.4%)	819 (8.9%)	541 (5.9%)	425 (4.6%)	278 (3.0%)	
Non-Hispanic White	3765 (40.9%)	1007 (10.9%)	1069 (11.6%)	986 (10.7%)	703 (7.6%)	
Other race	1371 (14.9%)	135 (1.5%)	271 (2.9%)	376 (4.1%)	589 (6.4%)	
Family PIR	3.10±0.05	3.22±0.07	3.12±0.06	3.10±0.07	2.94±0.07	0.002
Education level, %						0.250
Less than high school	1627 (17.7%)	405 (4.4%)	415 (4.5%)	420 (4.6%)	387 (4.2%)	
High school	2029 (22.0%)	573 (6.2%)	513 (5.6%)	485 (5.3%)	458 (5.0%)	
More than high school	5551 (60.3%)	1354 (14.7%)	1463 (15.9%)	1430 (15.5%)	1304 (14.2%)	
Marital status, %						0.450
Having a partner	5476 (59.5%)	1354 (14.7%)	1396 (15.2%)	1395 (15.2%)	1331 (14.5%)	
No partner	1913 (20.8%)	535 (5.8%)	500 (5.4%)	487 (5.3%)	391 (4.2%)	
Unmarried	1818 (19.7%)	443 (4.8%)	495 (5.4%)	453 (4.9%)	427 (4.6%)	
Smoker, %						0.002
Never	5288 (57.4%)	1215 (13.2%)	1314 (14.3%)	1370 (14.9%)	1389 (15.1%)	
Former	2197 (23.9%)	613 (6.7%)	606 (6.6%)	541 (5.9%)	437 (4.7%)	
Now	1722 (18.7%)	504 (5.5%)	471 (5.1%)	424 (18.2%)	323 (3.5%)	
Alcohol user, %						< 0.001
No	1189 (12.9%)	233 (2.5%)	258 (2.8%)	327 (3.6%)	371 (4.0%)	
Former	1093 (11.9%)	288 (3.1%)	273 (3.0%)	263 (2.9%)	269 (2.9%)	
Mild	3505 (38.1%)	934 (10.1%)	896 (9.7%)	894 (9.7%)	781 (8.5%)	
Moderate	1569 (17.0%)	376 (4.1%)	435 (4.7%)	395 (4.3%)	363 (3.9%)	
Heavy	1851 (20.1%)	501 (5.4%)	529 (5.7%)	456 (5.0%)	365 (4.0%)	
Hypertension, %						0.430
No	5253 (57.1%)	1212 (13.2%)	1343(14.6%)	1361 (14.8%)	1337 (14.5%)	
Yes	3954 (42.9%)	1120 (12.2%)	1048 (11.4%)	974 (10.6%)	812 (8.8%)	
DM, %						0.410
No	7500 (81.5%)	1836 (19.9%)	1987 (21.6%)	1902 (20.7%)	1775 (19.3%)	
Yes	1707 (18.5%)	496 (5.4%)	404 (4.4%)	433 (4.7%)	374 (4.1%)	
CHD, %						0.120
No	8867 (96.3%)	2223 (24.1%)	2306 (25.0%)	2247 (24.4%)	2091 (22.7%)	
Yes	340 (3.7%)	109 (1.2%)	85 (0.9%)	88 (1.0%)	58(0.6%)	
CHF, %						0.430
No	8942 (97.1%)	2250 (24.4%)	2327 (25.3%)	2262 (24.6%)	2103 (22.8%)	
Yes	265 (2.9%)	82 (0.9%)	64 (0.7%)	73 (0.8%)	46 (0.5%)	
Angina, %						0.790
No	9003(97.8%)	2278(24.7%)	2332 (25.3%)	2285 (24.8%)	2108 (22.9%)	
Yes	204 (2.2%)	54 (0.6%)	59 (0.6%)	50 (0.5%)	41 (0.4%)	
Heart attack, %						0.400
No	8860 (96.2%)	2225 (24.2%)	2296 (24.9%)	2244 (24.4%)	2095 (22.8%)	
Yes	347 (3.8%)	107 (1.2%)	95 (1.0%)	91 (3.9%)	54 (0.6%)	
Stroke, %						0.130
No	8880 (96.4%)	2216 (24.1%)	2311 (25.1%)	2263 (24.6%)	2090 (22.7%)	
Yes	327 (3.6%)	116 (1.3%)	80 (0.9%)	72 (0.8%)	59 (0.6%)	
PA, %						0.110
Never	5205 (56.5%)	1283 (13.9%)	1306 (14.2%)	1318 (14.3%)	1298 (14.1%)	
Mild	2079 (22.6%)	522 (5.7%)	564 (6.1%)	521 (5.7%)	472 (5.1%)	
Moderate	1578 (17.1%)	436 (4.7%)	428 (4.6%)	411 (4.5%)	303 (3.3%)	
Vigorous	345 (3.7%)	91 (1.0%)	93 (1.0%)	85(0.9%)	76 (0.8%)	
BMI, kg/m^2^	29.39 ± 0.15	28.47 ± 0.17	29.14 ± 0.20	29.92 ± 0.22	30.15 ± 0.29	< 0.001
waist circumference, cm	100.36 ± 0.35	99.28 ± 0.42	99.86 ± 0.52	101.37 ± 0.52	101.05 ± 0.72	< 0.001
SBP, mmHg	122.10 ± 0.32	122.86 ± 0.53	122.21 ± 0.51	122.05 ± 0.50	121.08 ± 0.46	0.050
DBP, mmHg	71.18 ± 0.32	71.09 ± 0.40	70.79 ± 0.46	71.34 ± 0.37	71.61 ± 0.37	0.100
Mean energy	2100.52 ± 12.69	2186.20 ± 24.29	2106.93 ± 19.32	2057.58 ± 24.55	2042.26 ± 23.42	< 0.001
intake (kcal/day)						
Hemoglobin, g/dL	14.21 ± 0.03	14.23 ± 0.04	14.33 ± 0.04	14.32 ± 0.04	13.90 ± 0.05	< 0.001
Glucose, mg/dl	99.66 ± 0.47	99.71 ± 0.76	99.42 ± 0.88	100.02 ± 0.85	99.46 ± 0.84	0.950
BUN, mg/dL	13.98 ± 0.10	14.53 ± 0.15	14.12 ± 0.16	13.90 ± 0.18	13.22 ± 0.17	< 0.001
UA, mg/dL	5.41 ± 0.02	5.51 ± 0.05	5.42 ± 0.04	5.42 ± 0.04	5.28 ± 0.04	0.004
Scr, mg/dL	0.88 ± 0.00	0.93 ± 0.01	0.89 ± 0.01	0.87 ± 0.01	0.83 ± 0.01	< 0.001
TC, mg/dL	193.67 ± 0.72	192.13 ± 1.26	194.12 ± 1.21	195.66 ± 1.28	192.41 ± 1.28	0.200
TG, mg/dL	151.42 ± 2.08	148.80 ± 3.10	155.83 ± 3.82	152.05 ± 3.10	148.03 ± 3.24	0.260
HDL-C, mg/dL	1.40 ± 0.01	1.41 ± 0.02	1.39 ± 0.01	1.38 ± 0.01	1.39 ± 0.02	0.540

*Abbreviations:* *Q1* 1.570–7.415 ug/L, *Q2* 7.416–9.230 ug/L, *Q3* 9.231–11.555 ug/L, *Q4* 11.556–57.770 ug/L, *Family PIR* family poverty-income ratio, *DM* diabetes mellitus, *CHD* coronary heart disease, *CHF* congestive heart failure, *BMI* body mass index, *SBP* Systolic blood pressure, *DBP* Diastolic blood pressure, *BUN* Blood urea nitrogen, *UA* Uric acid, *Scr* Serum creatinine, *TC* total cholesterol, *TG* triglyceride, *HDL-C* high density lipoprotein- cholesterol

### Correlation between manganese concentrations and sociodemographic and cardiovascular outcomes

**Fig. 1:**
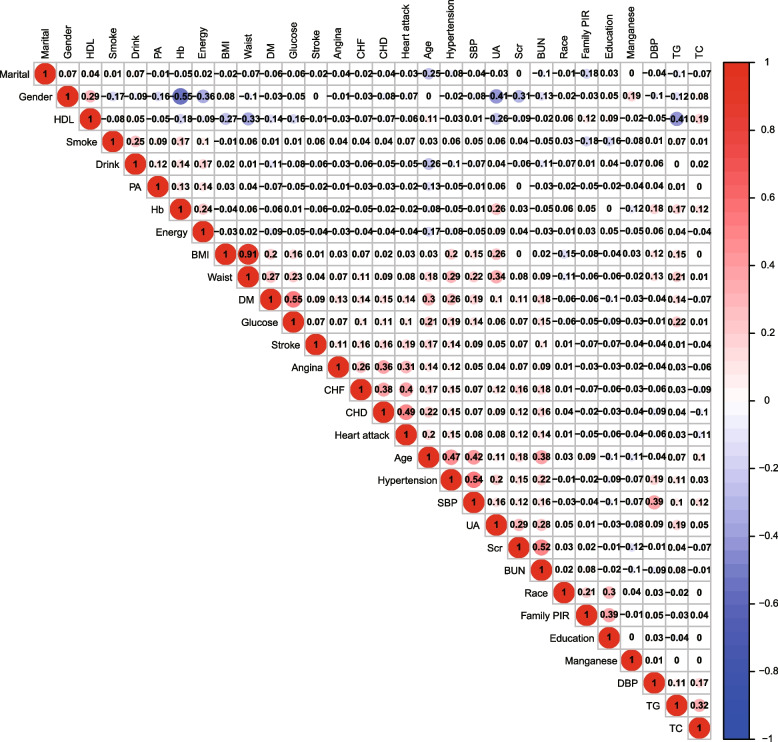
The association between Manganese and covariates. Abbreviation: family PIR, family poverty-income ratio; smoker, DM, diabetes mellitus; CHD, chronic heart disease; CHF, chronic heart failure; SBP, systolic blood pressure; DBP, diastolic blood pressure; BMI, body mass index; PA, physical activity; Hb, hemoglobin; BUN, blood urea nitrogen; UA, uric acid; Scr, serum creatinine; HDL-C, high density lipoprotein-cholesterol; TC, total cholesterol; TG, triglycerides

The correlation heatmap of manganese and covariates revealed that manganese was positively associated with sex (correlation coefficient, *r* = 0.19, *P* <0.001) and negatively associated with age (correlation coefficient, *r* =-0.11, *P* <0.001), and Scr (correlation coefficient, *r* = -0.12, *P* <0.001), respectively (Fig. 1).

### Association of manganese with all-cause, CVD-related and cancer-related mortality

**Fig. 2:**
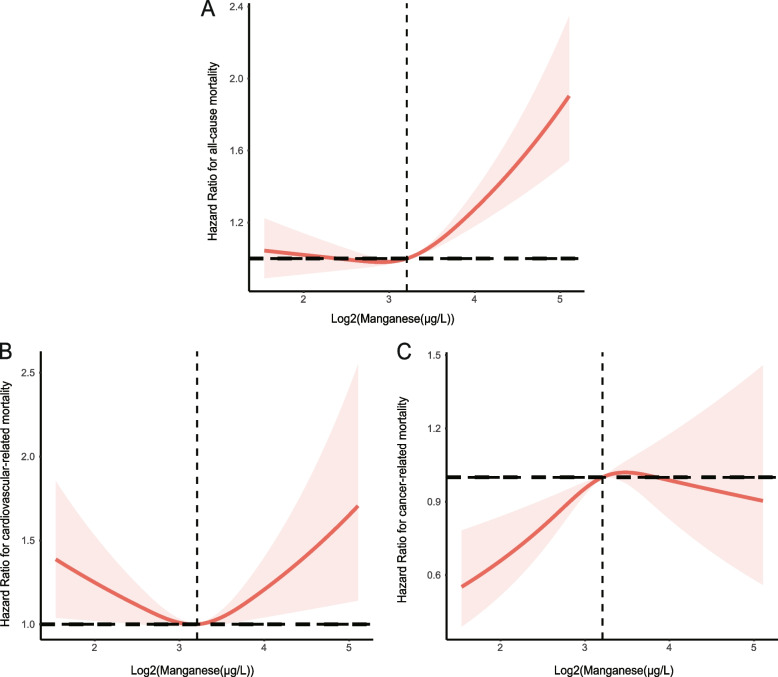
Restricted cubic spline plot of the association between serum Manganese concentration and **a** all-cause mortality, **b** CVD mortality, and **C** cancer-related mortality. Note: Manganese is log2 transformed. Solid and dashed lines represent the log-transformed odds ratios and the corresponding 95% confidence intervals. Abbreviation: CVD, Cardiovascular disease

The VIF values of age, sex, race/ethnicity, education level, family PIR, marital status, the complication of hypertension and DM, SBP, DBP, BMI, waist circumference, PA, mean energy intake, Hb, fast glucose, BUN, UA, Scr, TC, TG, and HDL-C were 1.431, 1.449, 1.322, 1.142, 1.095, 1.165, 1.235, 1.199, 1.284, 1.652, 1.164, 1.261, 1.227, 1.210, 2.678, 2.716, 1.014, 1.284, 1.145, 1.236, 1.619, 1.209, 1.448, 1.212, 1.275, and 1.363, respectively. In the fully adjusted model, compared with the first quartile of manganese exposure (Q1), the HRs (95% CIs) for all-cause and CVD-related mortality across the manganese quartiles were 1.11 (0.87, 1.41), 0.96 (0.74, 1.23), and 1.23 (0.96, 1.59), as well as 0.86 (0.54, 1.37), 0.87 (0.55, 1.40), and 1.07 (0.67, 1.72), respectively (Tables 2, and 3). Compared with subjects in Q1, the fully adjusted HRs (95% CIs) of cancer-related mortality were 1.493 (0.948, 2.351), 1.122 (0.686, 1.835), and 1.275 (0.757, 2.149) (Table 4). The restricted cubic spline (RCS) curve is shown in Figs. 2A and B, representing the U-curve relationship between manganese and all-cause and CVD-related mortality (*P* for nonlinearity <0.05). As serum manganese concentrations increased, the risk of all-cause mortality initially decreased significantly. The risk of all-cause mortality was lowest when serum manganese concentrations reached 7.47 µg/L, then the curve showed an upward trend. Meanwhile, when the concentration of serum manganese was <9.01 µg/L, CVD-mortality and serum manganese was a negative association. When the concentration of serum manganese was >9.01 µg/L, CVD-mortality and serum manganese was a positive association. There was also a nonlinear relationship between manganese and cancer-related mortality (Fig. 2C). When the serum manganese concentration increased, cancer-related mortality initially increased and then decreased.

**Table 2:** Adjusted HRs for associations between manganese and all-cause mortality

Manganese (ug/L)	Model 1	Model 2	Model 3
	HR (95%CI)	HR (95%CI)	HR (95%CI)
Q1	Ref.	Ref.	Ref.
Q2	0.88 (0.70, 1.11)	0.99 (0.78, 1.25)	1.11 (0.87,1.41)
Q3	0.79 (0.62, 1.01)	0.84 (0.66, 1.08)	0.96 (0.74, 1.23)
Q4	1.00 (0.79, 1.28)	1.11 (0.87, 1.43)	1.23 (0.96, 1.59)
*P* for trend	0.583	0.861	0.275

Model 1: age and gender. Model 2: model 1 variables plus race/ethnicity, education level, marital status, family poverty-income ratio, smoker, alcohol user, hypertension, diabetes mellitus. Model 3 was adjusted for model 2 variables plus chronic heart disease, chronic heart failure, angina, heart attack, stroke, systolic blood pressure, and diastolic blood pressure, body mass index, waist circumference, physical activity, mean energy intake, hemoglobin, glucose, blood urea nitrogen, uric acid, serum creatinine, high density lipoprotein-cholesterol, total cholesterol, and triglycerides.

*Abbreviations:* *Q1* 1.570–7.415 ug/L, *Q2* 7.416–9.230 ug/L, *Q3* 9.231–11.555 ug/L, *Q4* 11.556–57.770 ug/L, *HR* hazard ratio, *CI* confidence interval

**Table 3:** Adjusted HRs for associations between manganese and CVD-related mortality

Manganese (ug/L)	Model 1	Model 2	Model 3
	HR (95%CI)	HR (95%CI)	HR (95%CI)
Q1	Ref.	Ref.	Ref.
Q2	0.67 (0.43, 1.05)	0.78 (0.49, 1.22)	0.86 (0.54, 1.37)
Q3	0.72 (0.46, 1.13)	0.79 (0.50, 1.25)	0.87 (0.55, 1.40)
Q4	0.92 (0.59, 1.44)	1.05 (0.66, 1.67)	1.07 (0.67, 1.72)
*P* for trend	0.536	0.941	0.906

Model 1: age and gender. Model 2: model 1 variables plus race/ethnicity, education level, marital status, family poverty-income ratio, smoker, alcohol user, hypertension, diabetes mellitus. Model 3 was adjusted for model 2 variables plus chronic heart disease, chronic heart failure, angina, heart attack, stroke, systolic blood pressure, and diastolic blood pressure, body mass index, waist circumference, physical activity, mean energy intake, hemoglobin, glucose, blood urea nitrogen, uric acid, serum creatinine, high density lipoprotein-cholesterol, total cholesterol, and triglycerides.

*Abbreviations:* *Q1* 1.570–7.415 ug/L, *Q2* 7.416–9.230 ug/L, *Q3* 9.231–11.555 ug/L, *Q4* 11.556–57.770 ug/L, *HR* hazard ratio, *CI* confidence interval

**Table 4:** Adjusted HRs for associations between manganese and cancer-related mortality

Manganese (ug/L)	Model 1	Model 2	Model 3
	HR (95%CI)	HR (95%CI)	HR (95%CI)
Q1	Ref.	Ref.	Ref.
Q2	1.26 (0.80, 1.97)	1.35 (0.86, 2.13)	1.45 (0.92, 2.29)
Q3	1.03 (0.64, 1.67)	1.07 (0.66, 1.74)	1.14 (0.70, 1.88)
Q4	1.15 (0.69, 1.89)	1.21 (0.72, 2.03)	1.26 (0.75, 2.11)
*P* for trend	0.753	0.640	0.524

Model 1: age and gender. Model 2: model 1 variables plus race/ethnicity, education level, marital status, family poverty-income ratio, smoker, alcohol user, hypertension, diabetes mellitus. Model 3 was adjusted for model 2 variables plus chronic heart disease, chronic heart failure, angina, heart attack, stroke, systolic blood pressure, and diastolic blood pressure, body mass index, waist circumference, physical activity, mean energy intake, hemoglobin, glucose, blood urea nitrogen, uric acid, serum creatinine, high density lipoprotein-cholesterol, total cholesterol, and triglycerides

*Abbreviations:* *Q1* 1.570–7.415 ug/L, *Q2* 7.416–9.230 ug/L, *Q3* 9.231–11.555 ug/L, *Q4* 11.556–57.770 ug/L, *HR* hazard ratio, *CI* confidence interval

### Subgroups analysis for the associations of manganese with all-cause, CVD-related, and cancer-related mortality

Subgroup analyses for the associations of manganese with all-cause, CVD-related, and cancer-related mortality were conducted based on age, sex, race/ethnicity, hypertension, DM, CHD, CHF, angina pectoris, heart attack, stroke, and BMI (Tables 5, 6, and 7).

**Table 5:** Subgroups analysis for the associations of manganese with all-cause mortality

	Q1	Q2	Q3	Q4	*P* for trend	*P* for interaction
	HR (95%CI)	HR (95%CI)	HR (95%CI)	HR (95%CI)		
Age						0.164
< 60	1.00	0.85 (0.51, 1.42)	0.83 (0.50, 1.40)	1.14 (0.67, 1.93)	0.756	
≥ 60	1.00	1.15 (0.88, 1.51)	0.96 (0.72, 1.29)	1.18 (0.88, 1.58)	0.495	
Sex						0.014
Male	1.00	1.13 (0.84, 1.52)	1.00 (0.73, 1.37)	0.94 (0.65, 1.36)	0.762	
Female	1.00	0.93 (0.61, 1.40)	0.75 (0.48, 1.16)	1.43 (0.97, 2.09)	0.133	
Race						0.001
Mexican	1.00	1.28 (0.35, 4.73)	1.59 (0.48, 5.27)	1.83 (0.53, 6.35)	0.314	
American						
Other Hispanic	1.00	0.54 (0.12, 2.47)	0.74 (0.17, 3.20)	1.47 (0.32, 6.65)	0.508	
Non-Hispanic	1.00	0.86 (0.51, 1.45)	0.74 (0.41, 1.33)	0.85 (0.44, 1.63)	0.382	
Black						
Non-Hispanic	1.00	1.20 (0.88, 1.62)	1.02 (0.74, 1.41)	1.17 (0.84, 1.64)	0.525	
White						
Other race	1.00	12.81 (0.96, 170.55)	3.33 (0.22, 50.65)	12.26 (0.88, 170.84)	0.187	
Hypertension						0.092
No	1.00	0.95 (0.58, 1.55)	0.94 (0.56, 1.57)	1.11 (0.64, 1.91)	0.804	
Yes	1.00	1.17 (0.89, 1.54)	0.99 (0.74, 1.33)	1.33 (0.99, 1.79)	0.161	
DM						0.218
No	1.00	1.22 (0.90, 1.65)	0.93 (0.67, 1.31)	1.35 (0.97, 1.87)	0.243	
Yes	1.00	0.96 (0.64, 1.43)	0.96 (0.65, 1.42)	1.09 (0.71, 1.67)	0.794	
CHD						0.162
No	1.00	1.01 (0.78, 1.31)	0.94 (0.71, 1.23)	1.21 (0.92, 1.60)	0.323	
Yes	1.00	2.14 (1.04, 4.40) *	1.40 (0.68, 2.90)	1.69 (0.78, 3.68)	0.271	
CHF						0.007
No	1.00	1.03 (0.80, 1.34)	0.84 (0.63, 1.11)	1.24 (0.95, 1.63)	0.423	
Yes	1.00	1.95 (0.91, 4.18)	2.09 (1.01, 4.33)*	1.12 (0.48, 2.62)	0.437	
Angina pectoris						0.163
No	1.00	1.07 (0.84, 1.37)	0.96 (0.74, 1.25)	1.20 (0.92,1.56)	0.350	
Yes	1.00	1.04 (0.27, 4.06)	0.55 (0.13, 2.26)	2.13 (0.61, 7.48)	0.412	
Heart attack						0.033
No	1.00	1.01 (0.78, 1.30)	0.88 (0.67, 1.16)	1.19 (0.91, 1.57)	0.482	
Yes	1.00	2.41 (1.13, 5.16) *	2.45 (1.05, 5.71)	2.10 (0.88, 5.03)	0.071	
Stroke						0.153
No	1.00	1.04 (0.81, 1.34)	0.98 (0.75, 1.27)	1.21 (0.92, 1.59)	0.315	
Yes	1.00	1.57 (0.86, 2.88)	0.50 (0.25, 1.03)	1.66 (0.92, 2.97)	0.656	
BMI						0.134
< 30 kg m^2^	1.00	1.01 (0.74, 1.37)	1.02 (0.75, 1.40)	1.28 (0.93, 1.78)	0.194	
≥ 30 kg/m^2^	1.00	1.18 (0.81, 1.72)	0.82 (0.53, 1.27)	1.18 (0.77, 1.80)	0.865	

Analysis was adjusted for age, sex, race/ethnicity. education level, marital status, family poverty-income ratio, smoker, alcohol user, hypertension, diabetes mellitus, chronic heart disease, chronic heart failure, angina, heart attack, stroke, systolic blood pressure, diastolic blood pressure, body mass index, waist circumference, physical activity, mean energy intake, hemoglobin, glucose, blood urea nitrogen, uric acid, serum creatinine, high density lipoprotein-cholesterol, total cholesterol, and triglycerides

*Abbreviations: Q1* 1.570–7.415 ug/L, *Q2* 7.416–9.230 ug/L, *Q3* 9.231–11.555 ug/L, *Q4* 11.556–57.770 ug/L, *P < 0.05, *DM* diabetes mellitus, *BMI* body mass index, *HR* hazard ratio, *CI* confidence interval

**Table 6:** Subgroups analysis for the associations of manganese with CVD-related mortality

	Q1	Q2	Q3	Q4	*P* for trend	*P* for interaction
	HR (95%CI)	HR (95%CI)	HR (95%CI)	HR (95%CI)		
Age						0.006
< 60	1.00	0.81 (0.27, 2.41)	0.31 (0.07, 1.34)	2.32 (0.99, 5.47)	0.139	
≥ 60	1.00	0.83 (0.50, 1.37)	1.01 (0.61, 1.66)	0.82 (0.47, 1.42)	0.623	
Sex						0.163
Male	1.00	1.00 (0.56, 1.80)	0.79 (0.42, 1.48)	1.01 (0.51, 2.01)	0.754	
Female	1.00	0.65 (0.28, 1.49)	0.85 (0.40, 1.80)	1.13 (0.55, 2.30)	0.690	
Race						<0.001
Mexican	1.00	-	-	-	-	
American						
Other Hispanic	1.00	-	-	-	-	
Non-Hispanic	1.00	0.67 (0.26, 1.71)	0.39 (0.12, 1.22)	0.73 (0.22, 2.36)	0.213	
Black						
Non-Hispanic	1.00	0.70 (0.37, 1.32)	0.83 (0.45, 1.54)	0.81 (0.43, 1.53)	0.541	
White						
Other race	1.00	-	-	-	-	
Hypertension						0.004
No	1.00	0.14 (0.03, 0.73) *	0.52 (0.15, 1.75)	1.25 (0.39, 4.02)	0.986	
Yes	1.00	1.12 (0.67, 1.86)	1.01 (0.59, 1.71)	1.14 (0.67, 1.97)	0.718	
DM						0.022
No	1.00	0.76 (0.40, 1.44)	0.62 (0.30, 1.27)	1.16 (0.62, 2.16)	0.894	
Yes	1.00	1.05 (0.51, 2.13)	1.23 (0.63, 2.41)	0.89 (0.39, 2.01)	0.966	
CHD						0.019
No	1.00	0.61 (0.35, 1.07)	0.77 (0.45, 1.32)	1.06 (0.62, 1.80)	0.947	
Yes	1.00	3.11 (0.94, 10.33)	1.58 (0.47, 5.28)	1.78 (0.48, 6.52)	0.609	
CHF						0.021
No	1.00	0.68 (0.40, 1.15)	0.61 (0.35, 1.06)	0.95 (0.56, 1.63)	0.511	
Yes	1.00	1.68 (0.45, 6.21)	5.65 (1.59, 20.12)**	2.72 (0.68, 10.83)	0.045	
Angina pectoris						0.015
No	1.00	0.66 (0.40, 1.09)	0.84 (0.52, 1.37)	1.06 (0.65, 1.73)	0.863	
Yes	1.00	-	-	-	-	
Heart attack						0.005
No	1.00	0.66 (0.39, 1.11)	0.70 (0.41, 1.18)	1.01 (0.60, 1.69)	0.782	
Yes	1.00	3.39 (0.86, 13.45)	5.48 (1.26, 23.80)*	1.54 (0.33, 7.20)	0.391	
Stroke						0.003
No	1.00	0.68 (0.41, 1.13)	0.93 (0.57, 1.51)	0.86 (0.50, 1.49)	0.726	
Yes	1.00	1.92 (0.49, 7.57)	0.03 (0.01, 0.65)*	2.72 (0.62, 11.84)	0.313	
BMI						0.025
< 30 kg m^2^	1.00	0.60 (0.30, 1.19)	0.94 (0.52, 1.71)	0.89 (0.46, 1.73)	0.845	
≥ 30 kg/m^2^	1.00	1.35 (0.67, 2.70)	0.79 (0.34, 1.82)	1.51 (0.71, 3.19)	0.526	

Analysis was adjusted for age, gender, race/ethnicity. education level, marital status, family poverty-income ratio, smoker, alcohol user, hypertension, diabetes mellitus, chronic heart disease, chronic heart failure, angina, heart attack, stroke, systolic blood pressure, diastolic blood pressure, body mass index, waist circumference, physical activity, mean energy intake, hemoglobin, glucose, blood urea nitrogen, uric acid, serum creatinine, high density lipoprotein-cholesterol, total cholesterol, and triglycerides

*Abbreviations: Q1* 1.570–7.415 ug/L, *Q2* 7.416–9.230 ug/L, *Q3* 9.231–11.555 ug/L, *Q4* 11.556–57.770 ug/L, *P < 0.05; **P < 0.01, *CVD* cardiovascular disease, *DM* diabetes mellitus, *BMI* body mass index, *HR* hazard ratio, *CI* confidence interval

**Table 7:** Subgroups analysis for the associations of manganese with cancer-related mortality

	Q1	Q2	Q3	Q4	*P* for trend	*P* for interaction
	HR (95%CI)	HR (95%CI)	HR (95%CI)	HR (95%CI)		
Age						0.147
< 60	1.00	0.90 (0.30, 2.71)	1.11 (0.39, 3.19)	0.98 (0.32, 3.02)	0.930	
≥ 60	1.00	1.60 (0.96, 2.67)	1.11 (0.63, 1.98)	1.23 (0.68, 2.24)	0.683	
Gender						0.062
Male	1.00	1.77 (1.00, 3.15) *	1.37 (0.73, 2.56)	0.93 (0.42, 2.04)	0.867	
Female	1.00	1.08 (0.47, 2.45)	0.89 (0.38, 2.09)	1.55 (0.71, 3.37)	0.333	
Race						0.036
Mexican	1.00	-	-	-	-	
American						
Other Hispanic	1.00	1.87 (0.40, 8.69)	0.06 (0.01, 0.50)*	0.75 (0.14, 4.07)	0.361	
Non-Hispanic	1.00	1.35 (0.44, 4.14)	1.28 (0.35, 4.68)	0.58 (0.12, 2.82)	0.690	
Black						
Non-Hispanic	1.00	1.47 (0.83, 2.61)	1.31 (0.71, 2.41)	1.19 (0.60, 2.36)	0.588	
White						
Other race	1.00	-	-	-	-	
Hypertension						0.033
No	1.00	1.01 (0.43, 2.38)	0.94 (0.39, 2.27)	0.42 (0.13, 1.35)	0.210	
Yes	1.00	1.73 (0.99, 3.01)	1.18 (0.63, 2.19)	1.74 (0.95, 3.19)	0.172	
DM						0.043
No	1.00	1.96 (1.11, 3.46) *	1.22 (0.64, 2.32)	1.21 (0.61, 2.41)	0.843	
Yes	1.00	0.74 (0.29, 1.85)	1.07 (0.48, 2.42)	1.27 (0.55, 2.97)	0.526	
CHD						0.126
No	1.00	1.43 (0.88, 2.33)	1.24 (0.74, 2.07)	1.35 (0.78, 2.32)	0.345	
Yes	1.00	-	-	-	-	
CHF						0.013
No	1.00	1.68 (1.03, 2.76) *	1.31 (0.77, 2.22)	1.58 (0.92, 2.73)	0.172	
Yes	1.00	-	-	-	-	
Angina						0.084
No	1.00	1.51 (0.94, 2.43)	1.25 (0.76, 2.07)	1.30 (0.76, 2.22)	0.412	
Yes	1.00	-	-	-	-	
Heart attack						0.592
No	1.00	1.47 (0.90, 2.39)	1.11 (0.65, 1.87)	1.27 (0.73, 2.19)	0.579	
Yes	1.00	-	-	-	-	
Stroke						0.036
No	1.00	1.44 (0.90, 2.32)	1.10 (0.65, 1.84)	1.36 (0.80, 2.32)	0.408	
Yes	1.00	-	-	-	-	
BMI						0.399
< 30 kg m^2^	1.00	1.38 (0.76, 2.48)	1.28 (0.70, 2.35)	1.28 (0.66, 2.48)	0.443	
≥ 30 kg/m^2^	1.00	1.41 (0.65, 3.03)	0.94 (0.38, 2.34)	1.25 (0.51, 3.05)	0.841	

Analysis was adjusted for age, gender, race/ethnicity. education level, marital status, family poverty-income ratio, smoker, alcohol user, hypertension, diabetes mellitus, chronic heart disease, chronic heart failure, angina, heart attack, stroke, systolic blood pressure, diastolic blood pressure, body mass index, waist circumference, physical activity, mean energy intake, hemoglobin, glucose, blood urea nitrogen, uric acid, serum creatinine, high density lipoprotein-cholesterol, total cholesterol, and triglycerides

*Abbreviations: Q1* 1.570–7.415 ug/L, *Q2* 7.416–9.230 ug/L, *Q3* 9.231–11.555 ug/L, *Q4* 11.556–57.770 ug/L, *P < 0.05, *DM* diabetes mellitus, *BMI* body mass index, *HR* hazard ratio, *CI* confidence interval

## Discussion

Manganese is both a toxic and an essential trace element for human health and development. The present study examined the NHANES database and found that serum manganese concentration had a U-shaped association with all-cause, CVD-related, and cancer-related mortality in the American population. Monitoring and maintaining manganese concentrations at the low point of a U-shaped curve may help reduce all-cause, and CVD-related mortality.

Firstly, in our study, we found that there was the U-shaped relationship between manganese exposure and all-cause, CVD-related mortality in the American population. Manganese is toxic in low concentrations for several reasons. Manganese is an essential element that is involved in the synthesis and activation of many enzymes and in the regulation of the metabolism of glucose and lipids in humans. Judy L Aschner et al. found that manganese deficiency caused a number of detrimental effects, such as impaired growth, poor bone formation and skeletal defects, reduced fertility and birth defects, abnormal glucose tolerance, and altered lipid and carbohydrate metabolism in both animals and humans [20]. In addition, manganese deficiency is also associated with adverse metabolic and neuropsychiatric effects [21]. Previous studies only examined the association between manganese levels and all-cause mortality in the Chinese population and CVD-related mortality in the Japanese population [19, 22]. As a result of differences in selected observation endpoints, sample heterogeneity, sample size, and measurement equipment error, results differ. However, our study found that when manganese concentrations are maintained within a certain range (5.22–9.23 μg/L for all-cause mortality; 8.67–9.23 μg/L for CVD-related mortality), all-cause and CVD-related mortality decrease as manganese concentrations increase. Serum manganese concentrations in the range of 1.57–7.48 μg/L cause all-cause mortality to decrease as manganese concentrations increase. In addition, CVD-related mortality decreases as manganese concentrations increase, when serum manganese concentrations were between 1.57 and 9.03 μg/L. Researchers from China conducted a large-scale prospective clinical study involving 6,115 subjects to examine the association between 23 metal elements and all-cause mortality. Among them, manganese was found to be negatively correlated with all-cause mortality [22]. However, manganese exposure can be toxic to the human body, manifesting primarily in the nervous and cardiovascular systems. Zheng et al. found that manganese concentrations greater than >30 µM inhibit the contraction of the myocardium in different species, including rats, guinea pigs, dogs, conscious dogs, and rabbits [23]. This is consistent with the results of the U-shaped curve between manganese and CVD-related mortality we found. Additionally, Geir et al. have also demonstrated that manganese exposure adversely affects children's neurodevelopment when children ingest manganese in the drinking water at or above a level of 0.241 mg/L [24]. In a study based on a prospective cohort in Japan with a median follow-up of 16.5 years, 782 participants aged 40–79 years were found to have a lower cardiovascular-related mortality rate when their dietary manganese intake was increased (median from 3.0 mg/day to 10.0 mg/day in men; from 2.7 mg/day to 9.2 mg/day in women) [19]. Analysis of independent cardiovascular adverse events, including CHD, angina pectoris, heart attack, and stroke all revealed patterns commensurate with the general frequency of CVD. Cebi et al. recruited 30 CHD patients and 20 healthy controls and analyzed their plasma manganese content, and found no significant difference in plasma manganese concentration between the two groups [25]. We believed that this may be caused by the limited sample size. Heart attack is sometimes classified as acute myocardial infarction. Nowicki et al. assessed the relationship between the concentration of heavy metals (copper, zinc, manganese, cobalt, and iron) and the incidence of myocardial infarction by collecting the blood of patients with myocardial infarction (*n*=74) and the control population without myocardial infarction (*n*=72) and measuring the concentration of heavy metals in the blood [26]. They observed that manganese had the biggest area under the curve and had the strongest predictive value, with a substantial rise in the incidence of myocardial infarction related to higher manganese content. This is consistent with our result that the frequency of independent cardiovascular events rose dramatically after reaching the tipping threshold for blood manganese levels. In conclusion, due to its essential role in biological processes, a deficiency of Mn can lead to impaired biological functions, while an excess of this element is likely to be toxic [27].

Manganese can replace other metals in part of the enzyme action, without affecting its activity. Therefore, based on the interaction between metals, we considered that excessive manganese exposure plays a dominant role in the pathogenesis [10]. A stroke is a rapid, localized loss of neurological function caused by an infarction or bleeding in the brain, retina, or spinal cord. Weng et al. discovered in a case-control study that exposure to manganese was independently linked with the risk of stroke in the single-metal mode [28]. Secondly, we also revealed that when the serum manganese concentration increased, cancer-related mortality initially increased and then decreased. To date, evidence for a link between manganese and cancer has been scant, and conclusions have been inconsistent [29]. Li Z et al. revealed that manganese is significantly associated with incident cancer in the upper two quartiles. In addition, incidences of lung cancer showed a similar positive association [30]. Zhang Q et al. found a positive association between water manganese levels and cancer incidence, while Assem FL revealed a negative association between manganese levels (in tissue or serum) and hepatocellular carcinoma. These conflicting results may be a reflection of methodological variations (case-control and cohort studies) and differences in samples (water, serum, and plasma) [31, 32]. Finally, a significant U-curve association between manganese and all-cause mortality was also found among subjects who were female, with hypertension, and without DM. Female sex hormones, including estrogen and progesterone, and other physiological changes have an impact on the blood manganese concentration in women, which may have an impact on the preventive effect of manganese on CVD [33]. Additionally, manganese intake was inversely associated with CVD-related mortality in postmenopausal women [34]. In postmenopausal women, follicle-stimulating hormones regulate lipid metabolism and increase serum sex hormone-binding globulin levels, which protect against atherosclerotic CVD and its cardiometabolic risk factors [35]. Chen H and his team found that serum manganese concentration and diabetes have a U-shaped association in a Chinese population with hypertension [36]. This is consistent with our findings. Additionally, Shan Z et al. also revealed a similar U-shaped association between plasma manganese and DM in a case-control study conducted in the Chinese population [37]. Despite nonsignificant results, Wang X and his colleague conducted a cross-sectional study in Tianjin and found a U-shaped relationship between plasma manganese and DM [38].

There are several limitations to the study. To begin with, all of the included 9207 samples were drawn from the NHANES database. There were a sufficient number of subjects. However, manganese was largely derived from diet, and there were great differences in diet composition. Further prospective studies are required to determine whether the conclusions can be applied to the Chinese population and other international populations. Furthermore, as a cross-sectional study, recall and self-reporting bias might occur. Nevertheless, given these limitations, the need for more well-designed, multiple-centered trials in the future is critical to validating our findings.

## Conclusion

In summary, our results reveal that manganese and all-cause and CVD-related mortality were associated in a U-shaped relationship in the U.S. general population. Moderate intake of manganese can reduce all-cause mortality and CVD-related mortality. Further research is needed on the potential mechanism between manganese exposure and all-cause, and CVD-related mortality.

## Data Availability

The survey data are publicly available on the internet for data users and researchers throughout the world (www.cdc.gov/nchs/nhanes/).
